# Benign Aspirates on Follow-Up FNA May Be Enough in Patients with Initial Atypia of Undetermined Significance/Follicular Lesion of Undetermined Significance

**DOI:** 10.1155/2014/354612

**Published:** 2014-02-13

**Authors:** Ga Ram Kim, Jung Hyun Yoon, Eun-Kyung Kim, Hee Jung Moon, Jin Young Kwak

**Affiliations:** Department of Radiology and Research Institute of Radiological Science, Severance Hospital, Yonsei University College of Medicine, 50 Yonsei-ro, Seodaemun-gu, Seoul 120-752, Republic of Korea

## Abstract

*Background.* Management of thyroid nodules with benign aspirates following atypia of undetermined significance/follicular lesion of undetermined significance (AUS/FLUS) is not well established. We reviewed the risk of malignancy and the role of ultrasound (US) features among thyroid nodules with benign results following initial AUS/FLUS diagnoses. *Methods.* From December 2009 to February 2011, a total of 114 nodules in 114 patients diagnosed as benign on follow-up fine-needle aspiration (FNA) after AUS/FLUS results were included in our study. Eight among 114 nodules were confirmed pathologically and 106 were clinically observed by a follow-up FNA or US. Suspicious US features were defined as markedly hypoechogenicity, irregular or microlobulated margin, presence of microcalcifications, and taller than wide shape. *Results.* There were 110 (96.5%) benign nodules and 4 (3.5%) malignant nodules. Two (4.8%) among 42 nodules without suspicious US features and 2 (2.8%) out of 72 nodules with suspicious US features were confirmed as malignancy, but there were no significant associations between the malignancy rate and US features (*P* = 0.625). *Conclusion.* Clinical follow-up instead of surgical excision or continuous repeat FNA may be enough for benign thyroid nodules after AUS/FLUS. The role of US features might be insignificant in the management of these nodules.

## 1. Introduction

Fine-needle aspiration (FNA) is a useful test for thyroid nodule evaluation and has been widely accepted as the main diagnostic procedure [1–3]. In the past, no uniform criteria were established for the various diagnostic categories and diagnostic inconsistencies, a reason for difficulty in communicating the clinical implications of thyroid FNA results [4–6]. Fortunately, a uniform classification scheme was proposed with 6 distinct diagnostic categories in the 2007 National Cancer Institute Thyroid Fine Needle Aspiration State of the Science Conference and led to the subsequent Bethesda system for Reporting Thyroid Cytopathology [3, 7–9].

According to the Bethesda system, atypia of undetermined significance/follicular lesion of undetermined significance (AUS/FLUS) is a heterogeneous category. The risk of malignancy for all AUS/FLUS cases, including those patients with benign follow-up and in whom surgery was not performed, presumably is up to 5–15% and the recommendation for AUS/FLUS is repeat FNA follow-ups at appropriate intervals [8, 10]. Faquin and Baloch reported that no case was finally confirmed as malignancy among nodules with initial AUS/FLUS cytology results and benign results in repeat FNA [11]. They regarded repeat FNA as an effective means for definitive management-based diagnosis [11]. On the other hand, the rate of malignant diagnosis in patients with benign cytology results after an initial AUS/FLUS diagnosis was said to be 29% or 15%, in other studies, respectively [12, 13]. Since the risk of malignancy in patients with benign aspirates following an initial AUS/FLUS seems to remain higher than that of patients with a benign diagnosis alone, treating these patients in a equivalent manner is disputable and approaches to further management are not well established [12, 13].

In a recent study on thyroid nodules diagnosed as AUS/FLUS, the risk of malignancy was different according to ultrasound (US) findings, which provided ancillary information for patients with AUS/FLUS [14–16]. However, to our knowledge, the US features of nodules with benign results following initial AUS/FLUS cytology have not been examined. Therefore, in this study we reviewed the risk of malignancy and the role of US features among thyroid nodules with benign results following initial AUS/FLUS diagnoses.

## 2. Materials and Methods

### 2.1. Patients

Institutional review board approval was obtained and informed consent was waived for this retrospective study. Written informed consent was obtained from all patients for US-guided FNA prior to each procedure as part of daily clinical practice.

**Figure 1:**
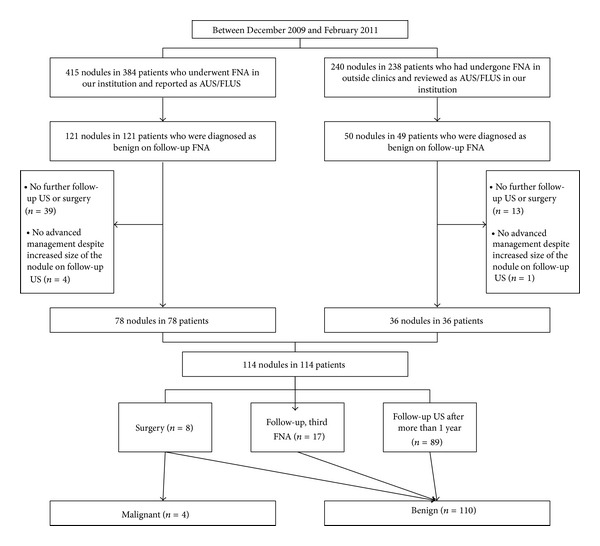
Flowchart of study population selection.* FNA: *fine needle aspiration; *AUS/FLUS:* atypia of undetermined significance/follicular lesion of undetermined significance.

From December 2009 to Feburuary 2011, a total of 6450 consecutive patients underwent US-guided FNA for 7954 thyroid nodules in our institution and reported as follows according to the Bethesda system: nondiagnostic or unsatisfactory (*n* = 1270, 16.0%), benign (*n* = 4664, 58.6%), atypia of undetermined significance or follicular lesion of undetermined significance (*n* = 415, 5.2%), follicular neoplasm or suspicious for follicular neoplasm (*n* = 63, 0.8%), suspicious for malignancy (*n* = 404, 5.1%), and malignant (*n* = 1138, 14.3%). Among 7954 nodules, 415 nodules (5.2%) in 384 patients were reported as AUS/FLUS. Of these, 121 nodules in 121 patients were diagnosed as benign on follow-up FNA. Among them, 43 nodules were excluded for the following reasons: no further follow-up including a second US-guided FNA or follow-up US after more than 1 year or no surgery (*n* = 39) and no advanced management such as surgery or additional FNA despite increased nodule size being noted on follow-up US (*n* = 4). A total of 78 nodules in 78 patients were included in this study. During the same period, based on the review of FNA slides by the pathology department of our institution, 240 nodules in 238 patients who had undergone US-guided FNA in outside clinics were classified as AUS/FLUS. Among them, 50 nodules in 49 patients were diagnosed as benign on follow-up US-guided FNA in our institution. Fourteen of 50 nodules without further follow-up (*n* = 13) and without additional work-up despite increase in size on follow-up US (*n* = 1) were excluded from this study. A total of 36 nodules in 36 patients with initial FNA done in outside hospitals were included in our study sample. Altogether, a total of 114 nodules in 114 patients were finally included in our study (Figure 1).

Of 114 nodules, 8 nodules were confirmed pathologically and 106 nodules were clinically observed by a third follow-up FNA (*n* = 17) or follow-up US after more than 1 year (*n* = 89). Surgery was performed in the patients for the following reasons: known papillary thyroid carcinoma in the other thyroid gland (*n* = 3), compression symptoms or cosmetic problems due to large goiter (*n* = 3), positive BRAF^V600E^ mutation on follow-up FNA (*n* = 2), patient's request to undergo surgery (*n* = 1), and a 3rd FNA which was “suspicious for papillary thyroid carcinoma” (*n* = 1). The mean period of follow-up US was 22.6 months (range, 12.0–36.4 months; median, 22.1 months). The 89 nodules observed by follow-up US showed decrease in size (*n* = 9) or no interval change in size (*n* = 80) on their follow-up US after more than 1 year.

### 2.2. US Exams, US-Guided FNA, and Cytologic Analysis

A 5–12 MHz linear probe (iU22, Philips Medical Systems, Bothell, WA) or a 6–13 MHz linear probe (EUB-7500, Hitachi Medical, Tokyo, Japan) was used for thyroid gland evaluation. Twelve board-certified radiologists specialized in thyroid imaging with 1–16 years of experience performed US and subsequent US-guided FNA. Markedly hypoechogenicity, irregular or microlobulated margin, presence of microcalcifications, and taller than wide shape were regarded as suspicious US features [17]. Size (the longest diameter of the nodule) and location (right or left thyroid gland and upper, mid, lower pole of thyroid gland) were also recorded.

US-guided FNA was performed by the same radiologist who performed the US. Each nodule was aspirated at least twice using the freehand technique with a 23-gauge needle connected with a 2-mL disposable plastic syringe. The specimen was expelled on a glass slide and then immediately placed in 95% alcohol for Papanicolaou staining [14, 18]. On-site evaluation was not performed in our institution. The Bethesda classifications were used in the cytology reports of thyroid aspirates.

### 2.3. Data and Statistical Analysis

We regarded cytopathological results as the “gold standard” and classified the cytopathological results as follows: cytopathologically confirmed malignancies were classified into the positive group and cytopathologically confirmed or clinical benign nodules through follow-up US were classified into the negative group. Comparisons of frequency distributions for categorical variables were performed by Fisher's exact test. Independent two-sample *t*-tests were used to compare continuous variables between benign and malignant nodules.

Analysis was performed using SPSS statistical software (SPSS Inc., Chicago, IL, ver 20.0), and statistical significance was accepted with *P* values <0.05.

## 3. Results

A total of 114 nodules from 114 patients (97 women, 17 men) were included. There were 110 (96.5%) benign nodules and 4 (3.5%) malignant nodules. The mean patient age was 49.9 ± 12.4 years (range, 17–77 years). The mean size of the 114 nodules was 13.5 ± 10.9 mm (range, 3–51 mm). The benign nodules were smaller than the malignant nodules, but without any statistical significance (*P* = 0.771, Table 1). There were no significant associations between the risk of malignancy and other baseline characteristics of nodules according to cytopathological outcomes (Table 1).

**Figure 2:**
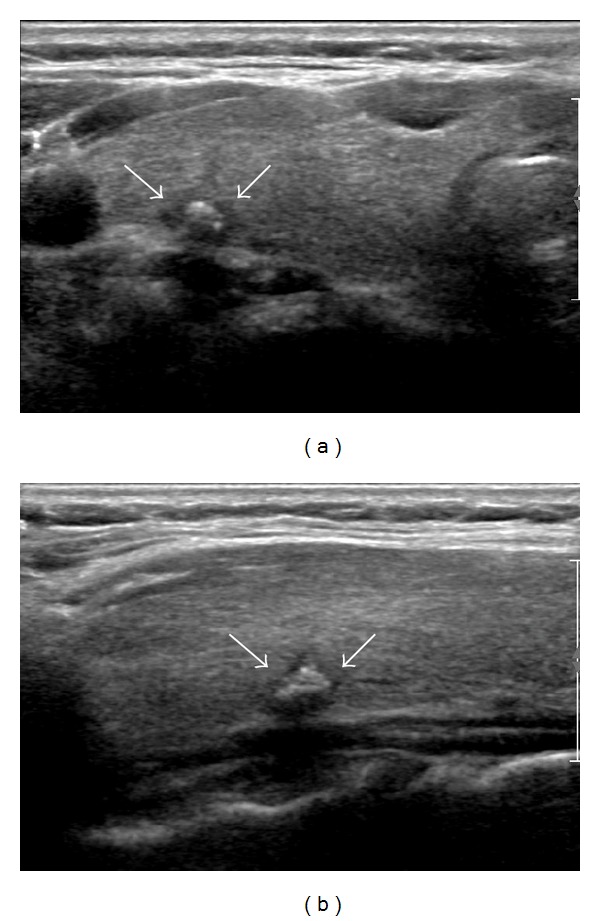
A 54-year-old woman diagnosed with papillary thyroid carcinoma. A 7 mm hypoechogenic nodule with a benign aspirate following an initial atypia of undetermined significance/follicular lesion of undetermined significance finding shows microlobulated margin and mixed calcifications on transverse US scan (a) and longitudinal US scan (b).

**Figure 3:**
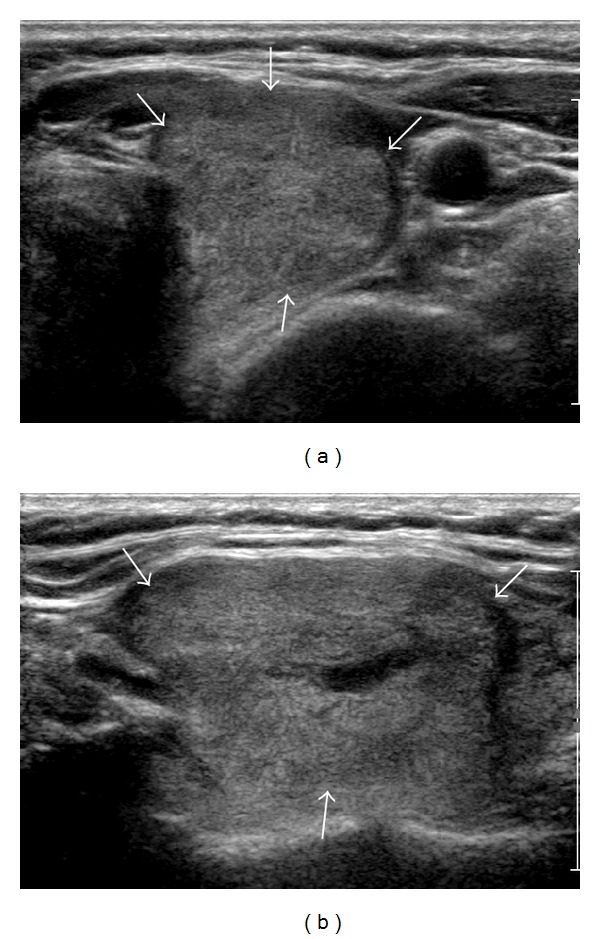
A 54-year-old woman diagnosed with follicular variant papillary thyroid carcinoma. A 43 mm isoechogenic mass with a benign aspirate following an initial atypia of undetermined significance/follicular lesion of undetermined significance finding shows no suspicious US features on transverse US scan (a) and longitudinal US scan (b).

US features of the 114 nodules are summarized in Table 2. Among the 114 nodules, 72 (63.2%) had no suspicious US features mentioned above whereas 42 (36.8%) showed one or more suspicious US features. Of the 72 nodules without suspicious US features, 2 (2.8%) were pathologically confirmed as malignancy. Of 42 nodules with suspicious US features, 2 (4.8%) were confirmed as malignancy. There was no statistical significant difference (*P* = 0.625). Four malignant nodules with benign aspirates following initial AUS/FLUS findings were pathologically confirmed by surgery (papillary thyroid carcinoma (*n* = 2), follicular variant papillary thyroid carcinom (*n* = 1), and follicular carcinoma (*n* = 1)) (Table 3; Figures 2 and 3). All 4 patients were confirmed with euthyroid status without thyroid autoantibodies. Among the 110 nodules diagnosed as benign, four were pathologically confirmed as benign by surgery (adenomatous hyperplasia (*n* = 2) and follicular adenoma (*n* = 2)). The other 106 nodules were clinically observed by follow-up FNA (*n* = 17) or follow-up US (*n* = 89). Out of 17 nodules with benign follow-up aspirates, 15 nodules were reported as benign follicular nodules and 2 nodules were lymphocytic thyroiditis.

## 4. Discussion

The rate of AUS/FLUS has varied from 1.2% to 17.8% although the Bethesda system limits its designation to approximately 7% or fewer of all thyroid FNAs and an effort should be made to assign this category as little as possible [8, 19–23]. The appropriate management of AUS/FLUS is a great challenge as the category describes a wide variety of scenarios due to lack of strict cytological morphologic criteria. Even though surgery might definitively resolve clinical uncertainty, unnecessary surgery should be avoided when possible considering that most patients have been proven to have benign diseases [2, 24]. Before the Bethesda system, thyroid lesions which did not fit into the benign or malignant category were placed into an “indeterminate” category [25]. Most patients with this category were referred for diagnostic surgery, but the majority proved to have benign disease, which meant that unnecessary surgeries had been mostly performed [24, 26]. After the indeterminate category was split by the Bethesda system, repeat FNA in patients diagnosed as AUS/FLUS clearly allowed better selection of patients needing surgical excision [11, 27, 28]. Patients with repeat FNA after AUS/FLUS diagnosis underwent surgical excision more frequently than patients without repeat FNA after AUS/FLUS diagnosis and the malignancy rate on surgical excision was also different between the two groups [11]. Moreover, because many malignancies diagnosed after an AUS/FLUS aspiration are well-differentiated tumors with little chance of progression and as a large majority of thyroid cancers show indolent behavior, surgery might as well be reserved for therapeutic purposes [13, 29–32].

The recommended approach to initial AUS/FLUS is repeat FNA which can result in a more definitive diagnosis for about 80% of the nodules based on the Bethesda system [8, 23]. However, it is controversial whether a thyroid nodule with a benign result on repeat FNA after AUS/FLUS diagnosis should be conservatively managed like a thyroid nodule with benign cytology alone [11, 18]. The malignancy risk of a benign aspirate after an initial AUS/FLUS diagnosis is higher than 15% in some studies [12, 13]. On the other hand, in some studies, the malignancy rate in nodules with a benign aspirate after an initial AUS/FLUS diagnosis was much lower than 3% which is even lower than the assumed malignancy rate of the benign category in the Bethesda system [11, 27]. This variability in the reported malignancy rates of AUS/FLUS reflects the subjectivity and heterogeneity of this category [33, 34]. In this study, we demonstrated that 4 (3.5%) out of 114 nodules with benign aspirates following initial AUS/FLUS were diagnosed as malignant nodules. This malignancy rate of 3.5% is similar to that for patients diagnosed as benign according to the Bethesda system for thyroid cytopathology (assumed malignancy rate in the 0–3% range) for whom clinical follow-up is typically recommended as the usual management [8]. Accordingly, it is evident that clinical and radiological follow-up instead of early surgical intervention can be suggested carefully in the nodules with benign aspirates following initial AUS/FLUS results in view of the presented results of our study as well as other previous studies [11, 27, 28].

US features of thyroid nodules with AUS/FLUS will help differentiate malignancy. Although AUS/FLUS nodules without suspicious US features cannot be managed conservatively exactly like benign nodules, nodules with suspicious US features showed a high malignancy rate ranging from 41.7% to 100%, leading to direct surgery without treatment delay [14–16]. Even nodules having the same FNA results revealed different malignancy rates depending on their US features [35]. We intended to discover the role of US features of benign aspirate results after an initial AUS/FLUS or predictive US features for malignancy in nodules with benign results after AUS/FLUS cytology. There were no significant differences in clinical characters or US features between malignant and benign nodules: half of the 4 nodules confirmed as cancer had no suspicious US features and the other 2 nodules showed suspicious US features.

Our study has several limitations. First, our sample size is relatively small; therefore, a further study with a large sample size is needed to establish the most appropriate management and find occult clinical implications of US findings in this group. Second, not all nodules were confirmed through surgery, but this limitation may be inevitable because not all thyroid nodules detected on US undergo surgery in clinical practice. Third, the statistical power about the differentiation of US features according to malignancy in the study sample may be compromised because the number of cases confirmed as malignancy in our study was too small. Fourth, interobserver and intraobserver variability was not considered when US findings of thyroid nodules were assessed or when cytological results were found as AUS/FLUS. However, in previously published studies, experienced radiologists performing US guided FNA in our institution have shown substantial agreement using Cohen's kappa statistics and their final assessments have been proven to be highly accurate [36].

In conclusion, our study suggests that benign aspirates on follow-up FNA in patients with initial AUS/FLUS diagnoses may be enough to recommend clinical follow-up instead of surgical excision or continuous repeat FNA. The role of US features was insignificant in the management of this group, but further studies with larger samples are required.

## Figures and Tables

**Table 1:** Demographic and baseline characteristics in 114 patients with 114 thyroid nodules with benign aspirates following initial AUS/FLUS.

Characteristics	Standard reference		*P* value
	Benign (*n* = 110)	Malignant (*n* = 4)	
Gender			>0.999
Male	17 (15.5%)	0	
Female	93 (84.5%)	4 (100%)	
Age (years)	49.9	49.0	0.900
Size (mm)	13.5	16.0	0.797
Location			0.304
Right	66 (60.0%)	1 (25.0%)	
Left	44 (40.0%)	3 (75.0%)	
Location			0.074
Upper pole	23 (20.9%)	0	
Mid pole	45 (40.9%)	4 (100%)	
Lower pole	42 (38.2%)	0	
Underlying thyroid echogenicity			0.195
Homogenous	88 (80.0%)	2 (50.0%)	
Heterogenous	22 (20.0%)	2 (50.0%)	
Multiplicity			0.630
Single	49 (44.5%)	1 (25.0%)	
Multiple	61 (55.5%)	3 (75.0%)	

AUS/FLUS: atypia of undetermined significance/follicular lesion of undetermined significance.

**Table 2:** Ultrasound features of 114 lesions.

US features	Standard reference		*P* value
	Benign (*n* = 110)	Malignancy (*n* = 4)	
Composition			0.412
Solid	97 (88.2%)	3 (75.0%)	
Mixed, solid < 50%	5 (4.5%)	0	
Mixed, solid > 50%	8 (7.3%)	1 (25.0%)	
Echogenicity			0.472
Hyperechogenicity	2 (1.8%)	0	
Isoechogenicity	44 (40.0%)	3 (75.0%)	
Hypoechogenicity	59 (53.6%)	1 (25.0%)	
Markedly hypoechogenicity	5 (4.5%)	0	
Margin			>0.999
Well-defined	66 (60.0%)	3 (75.0%)	
Microlobulated	37 (33.6%)	1 (25.0%)	
Irregular	7 (6.4%)	0	
Calcifications			0.562
Microcalcifications	11 (10.0%)	1 (25.0%)	
Macrocalcifications	9 (8.2%)	0	
No calcification	90 (81.8%)	3 (75.0%)	
Shape			0.436
Wider than tall	96 (87.3%)	3 (75.0%)	
Taller than wide	14 (12.7 %)	1 (25.0%)	
Vascularity			>0.999
Peripheral	38 (34.5%)	2 (50.0%)	
Central	1 (0.9%)	0	
Both peripheral and central	39 (35.5%)	1 (25.0%)	
No vascularity	32 (29.1%)	1 (25.0%)	
US assessment			0.625
Without suspicious US features	70 (63.6%)	2 (50.0%)	
With one or more suspicious US features	40 (36.4%)	2 (50.0%)	

**Table 3:** Pathologically confirmed malignancy cases (*n* = 4).

Case number	Sex	Age	Family history	Physical examination	Tumor Size, location	Presence of suspicious US features	Pathologic diagnosis	Type of operation	Postoperative TMN staging	Recurrence	Disease-free time interval (from surgery to the latest US, day)
1	Female	57	None	Not specific	6 mm, left	No	PTC	Total thyroidectomy	T3N0	No	993
2	Female	30	None	Left palpable neck mass	43 mm, left	No	FVPTC	Total thyroidectomy	T3N0	No	33
3	Female	54	None	Not specific	7 mm, right	Yes	PTC	Hemithyroidectomy	T1N0	No	773
4	Female	55	None	Not specific	8 mm, left	Yes	FC	Right total and left partial thyroidectomy	T1N0	No	785

PTC: papillary thyroid carcinoma; FVPTC: follicular variant papillary thyroid carcinoma; FC: follicular carcinoma.
